# NEPA (Netupitant/Palonosetron) for the Prevention of Chemotherapy‐Induced Nausea and Vomiting (CINV) in Patients Receiving Highly or Moderately Emetogenic Chemotherapy Who Experienced Breakthrough CINV in Cycle 1 of Chemotherapy: A Phase II Clinical Trial

**DOI:** 10.1002/cam4.70549

**Published:** 2025-03-27

**Authors:** Rudolph M. Navari, Erminio Bonizzoni

**Affiliations:** ^1^ World Health Organization Mount Olive Alabama USA; ^2^ RIDE2Med Foundation Milan Italy

**Keywords:** breakthrough CINV, NEPA, netupitant, olanzapine

## Abstract

**Background:**

Although control of chemotherapy‐induced nausea and vomiting (CINV) is substantially improved with guideline‐directed antiemetic prophylaxis, breakthrough CINV remains a significant clinical patient problem. In subsequent cycles after breakthrough occurs, antiemetic guidelines recommend adding agents not used in the initial cycle. This study was designed to evaluate the use of NEPA (netupitant/palonosetron) plus dexamethasone with or without olanzapine for the prevention of CINV in the second cycle of chemotherapy for patients receiving highly (HEC) or moderately emetogenic chemotherapy (MEC) who developed breakthrough CINV in their first cycle despite guideline‐directed prophylactic antiemetics.

**Methods:**

This was a Phase 2, single center, open‐label study. Patients received guideline‐recommended prophylactic antiemetics in Cycle 1 based on the chemotherapy emetogenicity. Patients who experienced breakthrough CINV in Cycle 1 received intravenous (IV) NEPA (Day 1) plus dexamethasone (Days 1–4) and olanzapine (Days 1–4) for HEC or IV NEPA (Day 1) plus dexamethasone (Days 1–4) for MEC in Cycle 2.

**Results:**

Of the 227 patients enrolled in Cycle 1, 100 patients (*n* = 37 HEC, 63 MEC) experienced breakthrough CINV and received the NEPA‐based treatments in Cycle 2. The complete response (no emesis/no rescue use) rates [95% confidence intervals] during the overall (0–120 h) phase were 76% [59%, 88%] and 79% [67%, 89%] in the HEC and MEC groups, respectively.

**Conclusion:**

These results show that NEPA with or without olanzapine is an effective approach for CINV prevention for patients receiving HEC or MEC who develop breakthrough CINV after their first course of chemotherapy. The results support the antiemetic guideline recommendations.

**Trial Registration:**

clinicaltrials.gov identifier: NCT06065722

## Graphical abstract

This study was designed to evaluate the use of NEPA (netupitant/palonosetron) plus dexamethasone with or without olanzapine for the prevention of CINV in the second cycle of chemotherapy for patients receiving highly (HEC) or moderately emetogenic chemotherapy (MEC) who developed breakthrough CINV in their first cycle despite guideline‐directed prophylactic antiemetics. Of the 227 patients enrolled in Cycle 1, 100 patients (*n* = 37 HEC, 63 MEC) experienced breakthrough CINV and received the NEPA‐based treatments in Cycle 2. The complete response (no emesis/no rescue use) rates during the overall (0–120 h) phase were 76% and 79% in the HEC and MEC groups, respectively, suggesting that NEPA with or without olanzapine is an effective approach for CINV prevention for patients receiving HEC or MEC who develop breakthrough CINV after their first course of chemotherapy.

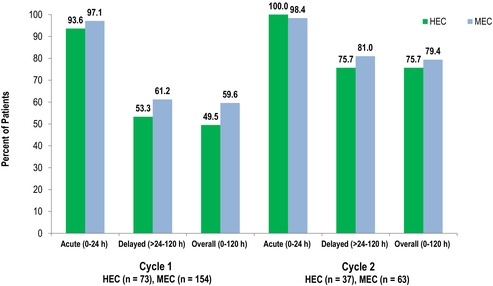

## 1 Introduction

Chemotherapy‐induced nausea and vomiting (CINV) has a negative impact on patients' quality of life and may influence their treatment decisions [1]. When CINV is not well controlled, it can lead to patients returning to the chemotherapy facility after treatment for rehydration or to manage vomiting and nausea. If outpatient facilities cannot effectively manage CINV, patients might need to seek care in an emergency department or be hospitalized [1]. CINV has been recognized by Medicare as a contributing factor to avoidable acute care. A recent study found that patients treated with highly emetogenic chemotherapy (HEC) had high rates of avoidable acute care related to CINV [2]. The risk of CINV is influenced by factors such as the emetogenicity of the chemotherapy administered, prior experience with CINV, and specific patient characteristics like gender, age, and history of low alcohol intake [1, 3].

The prevention of CINV has significantly improved because of the introduction of 5‐hydroxytryptamine‐3 (5‐HT3) receptor antagonists (RA) [4, 5]. Further enhancement has occurred with the use of neurokinin‐1 (NK‐1) RAs [6, 7], as well as olanzapine, an antipsychotic that blocks multiple neurotransmitters in the central nervous system [8, 9, 10, 11, 12, 13].

The optimal combinations of effective antiemetic agents in various clinical settings have been described in established guidelines from the Multinational Association of Supportive Care in Cancer (MASCC) and the European Society of Medical Oncology (ESMO) [14, 15, 16], the American Society of Clinical Oncology (ASCO) [17], and the National Comprehensive Cancer Network (NCCN) [18]. However, even with guideline‐based antiemetic prophylaxis and improved control of CINV, breakthrough CINV remains a significant clinical challenge [1, 19]. When breakthrough CINV occurs, immediate treatment may be considered with agents such as dopamine antagonists, olanzapine, metoclopramide, or benzodiazepines [1, 14, 17, 18]. The aforementioned guidelines [14, 17, 18] suggest treating breakthrough CINV with an agent from a drug class that was not used in the prophylactic regimen and recommend continuing the breakthrough medication until the nausea and vomiting is controlled. In subsequent chemotherapy cycles after breakthrough has occurred, the guidelines generally suggest adding to the previous prophylactic regimen an agent from a different drug class that was not used in the prior chemotherapy cycle (e.g., an NK‐1 RA or olanzapine). Notwithstanding these recommendations, there have been very few clinical trials evaluating the effectiveness of treatments immediately following breakthrough CINV [13, 19, 20, 21, 22] or in managing CINV in the subsequent cycle of chemotherapy following breakthrough CINV [23, 24].

Studies have shown beneficial effects in immediately treating breakthrough nausea and vomiting with oral prochlorperazine or an oral 5‐HT3 RA [20], a transdermal gel consisting of lorazepam, diphenhydramine, and haloperidol [21] and olanzapine [13, 22].

Although these trials demonstrated effective rescue agents for use in treating breakthrough CINV, patient response to a different antiemetic regimen during the subsequent chemotherapy cycle following failure in the first cycle is of clinical interest and aligned with those guidelines that make recommendations regarding the next cycle [18]. There are only two studies of this nature that we are aware of [23, 24]. One study evaluated changing 5‐HT3 RA agents following breakthrough CINV [23] and showed that CINV control may be enhanced if the antiemetic regimen is changed in the subsequent cycle, even by simply changing agents within the same class of drugs. The other study showed a beneficial effect of adding an NK1 RA in the subsequent cycle following breakthrough CINV in patients who received prophylaxis with a 5‐HT3 RA and dexamethasone prior to carboplatin‐based chemotherapy in Cycle 1 [24].

Additional studies are needed to confirm the value of switching agents within the same antiemetic class of drugs after breakthrough CINV, but more importantly, to explore the CINV control rates in subsequent chemotherapy cycles after adding to the existing antiemetic regimen, per the established guideline recommendations [14, 17, 18]. The international antiemetic guidelines [14, 17, 18] recommend adding a NK‐1 RA or olanzapine to the prophylactic regimen if not used in the initial cycle. The purpose of the current study was to evaluate the use of NEPA (netupitant/palonosetron) plus dexamethasone with or without olanzapine for the prevention of CINV in the second cycle of chemotherapy for patients receiving HEC or MEC who developed breakthrough CINV in their first cycle of chemotherapy despite the use of guideline‐directed prophylactic antiemetics.

NEPA is the only fixed antiemetic combination and is comprised of an NK1 RA (netupitant [oral] or fosnetupitant [intravenous]) and the 5‐HT3 RA, palonosetron [25]. The simultaneous targeting of two critical antiemetic pathways with the single dose administration results in simplified, efficient dosing. In clinical trials NEPA + dexamethasone showed superior prevention of CINV compared to palonosetron + dexamethasone [26, 27, 28, 29] and some evidence of potential benefits over an aprepitant regimen in the latter part of the delayed phase [30, 31] and during the extended overall phase [32]. Emerging data on olanzapine suggests a compelling additive effect when used in combination with an NK1 RA regimen [11].

Expanding on the small study by Valerio et al. [24], this study not only explores the efficacy of NEPA in patients who experienced breakthrough CINV in the prior cycle of either HEC or MEC, but also importantly, this is the first study to evaluate NEPA in combination with olanzapine.

## 2 Methods

### 2.1 Study Design

This was a Phase 2, single center, open‐label study conducted between October 2020 and January 2022. The trial protocol was approved by the institutional ethics committee, all patients provided written informed consent prior to initiation of any study treatment, and the study was conducted in accordance with recognized international scientific and ethical standards, including but not limited to the International Conference on Harmonization guideline for Good Clinical Practice (ICH GCP) and the Declaration of Helsinki. This study was registered on clinicaltrials.gov.

The primary objective was to assess CINV control as measured by the complete response (CR: no emetic episodes and no use of rescue medications) during the 5 days post chemotherapy (overall phase) in patients receiving NEPA plus dexamethasone with or without olanzapine in Cycle 2 for those patients receiving HEC or MEC who developed breakthrough CINV during Cycle 1.

### 2.2 Patients

Eligible patients were adult (≥ 18 years) males or females, naïve to chemotherapy, and scheduled to receive their first course of either HEC or MEC for treatment of a solid malignant tumor. Patients were required to have an Eastern Cooperative Oncology Group (ECOG) Performance Status of 0 or 1. Patients were ineligible if they experienced nausea or vomiting within the 24 h prior to enrollment/chemotherapy, if they were pregnant, if they had any known history of active, untreated CNS disease, cardiac arrythmia, congestive heart failure, or diabetes mellitus, or were receiving concurrent abdominal radiotherapy or concurrent olanzapine.

### 2.3 Treatments

**FIGURE 1:**
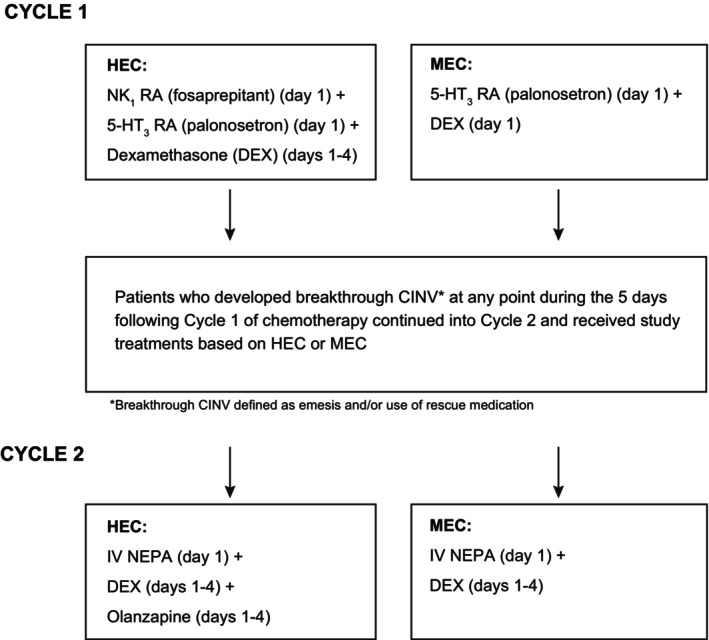
Study schema and treatments administered.

Patients enrolled into the study received prophylactic antiemetics in Cycle 1 per guideline recommendations based on the emetogenicity of the chemotherapy being administered (i.e., a triple NK1 RA (fosaprepitant)/5‐HT3RA (palonosetron)/dexamethasone regimen for HEC and a 5‐HT3 RA (palonosetron)/dexamethasone doublet for MEC) (Figure 1) [14, 17, 18]. NEPA use was excluded in Cycle 1. Patients who experienced breakthrough CINV in Cycle 1 received the study treatments in Cycle 2 as follows. Patients receiving HEC were administered IV NEPA (235 mg fosnetupitant/0.25 mg palonosetron) plus dexamethasone (12 mg IV) and olanzapine (5 mg PO) prior to chemotherapy on Day 1 plus dexamethasone (8 mg PO) and olanzapine (5 mg PO) on Days 2–4. The NCCN Antiemetic Guidelines [18] recommend olanzapine 5 or 10 mg daily for 4 days based on the efficacy reported in 2 recent studies [10, 33]; the 5 mg dose was selected to minimize any potential toxicity. Patients receiving MEC were administered IV NEPA and dexamethasone (8 mg IV) prior to chemotherapy on Day 1 and dexamethasone (4 mg PO) on Days 2–4. All patients received the IV antiemetic treatments in both Cycles 1 and 2 via a central line or port; no peripheral lines were used.

### 2.4 Assessments

From the start of chemotherapy on Day 1 in both Cycles 1 and 2 until the morning after Day 5 (0–120 h), each patient completed daily questionnaires capturing emetic episodes, severity of nausea and concomitant medications taken for the prior 24 h. An emetic episode was defined as any episode of vomiting or retching or combined vomiting and retching with separate episodes defined as occurring at least 1 min apart. Severity of nausea was evaluated daily using a 10‐point scale, with 0 reflecting “no nausea at all” and 10 reflecting “nausea as bad as it can be”. Patients were permitted to take rescue therapy of the investigator's choice for nausea and/or emesis/retching, based on clinical circumstances. As the patient's oncologist or primary care provider could also prescribe rescue medication as needed, specific details on type of rescue medication were not captured.

The primary efficacy endpoint in the study was complete response (no emesis and no use of rescue medication) during the overall (0–120 h) phase following initiation of chemotherapy. Complete response, defined as such, is viewed as the “gold standard” endpoint in antiemetic trials. Secondary endpoints included complete response rates during the acute (0–24 h) and delayed (> 24–120 h) phases. Complete response is reported for the total population in Cycles 1 and 2 and by gender as a post hoc assessment. Although complete response includes no use of rescue medication, an objective surrogate for no nausea, because patients are allowed to take rescue medication if they experience nausea, additional secondary endpoints included the proportion of patients with no nausea as well as the median nausea scores in those patients who experienced any level of nausea. Safety was assessed through collection of treatment‐related adverse events.

### 2.5 Statistical Analysis

Sample size was computed according to a single‐group design and based on the overall (0–120 h) complete response rates in Cycle 2 for the subgroup of patients who developed breakthrough CINV during Cycle 1. For a null hypothesis of a complete response rate of 20% versus the alternative hypothesis of a complete response rate of 40% (based on the results of the Arevalo–Aurajo study [23]) with two‐sided *α* = 0.05 and power = 0.80, a sample size of 44 patients was needed. Allowing for a 10% patient drop out, 50 patients in the MEC group and 50 patients in the HEC group was planned. However, the actual patients continuing into Cycle 2 of the study exceeded the planned (*n* = 63) in the MEC group and was lower than planned (*n* = 37) in the HEC group.

For each of the observation periods (acute, delayed and overall), the complete response rate for the HEC and MEC groups were estimated as a binomial proportion. A two‐sided 95% confidence interval for the sample proportion was estimated for the total population in Cycle 2 using the exact binomial (Clopper–Pearson) [34] method.

Descriptive statistics were used to summarize demographics, baseline characteristics, treatment‐related adverse events (AEs), serious AEs, and AEs that lead to discontinuation from study treatment.

## 3 Results

### 3.1 Patient Characteristics

Two hundred twenty‐seven patients entered Cycle 1 of the study and received the protocol‐specified guideline‐recommended antiemetic prophylaxis based on whether they were to receive HEC or MEC (Figure 1). Those in the HEC group (*n* = 73) were either patients with breast cancer receiving anthracycline/cyclophosphamide (AC) chemotherapy or patients with non‐small cell lung cancer (NSCLC) receiving cisplatin‐based chemotherapy (Table 1). The MEC group (*n* = 154) consisted predominantly of patients with colorectal cancer (*n* = 93; 60%). Other cancer types included pancreatic, gastric, bladder, appendix, and rectal. Almost all MEC patients (*n* = 145; 94%) received folinic acid, fluorouracil, and oxaliplatin (FOLFOX) chemotherapy; the remaining received gemcitabine/paclitaxel (*n* = 9).

**TABLE 1:** Baseline characteristics—patients entering Cycles 1 and 2.

Characteristic	Cycle 1		Cycle 2	
	HEC (*N* = 73)	MEC (*N* = 154)	HEC (*N* = 37)	MEC (*N* = 63)
Gender				
Male	30 (41.1%)	75 (48.7%)	12 (32.4%)	37 (58.7%)
Female	43 (58.9%)	79 (51.3%)	25 (67.6%)	26 (41.3%)
Age range (years)				
51–60	16 (21.9%)	39 (25.3%)	7 (18.9%)	12 (19.0%)
61–70	25 (34.2%)	66 (42.9%)	13 (35.1%)	25 (39.7%)
71–80	20 (27.4%)	39 (25.3%)	8 (21.6%)	23 (36.5%)
81–90	12 (16.4%)	10 (6.5%)	9 (24.3%)	3 (4.8%)
Race				
Caucasian	45 (61.6%)	89 (57.8%)	15 (40.5%)	30 (47.6%)
African American	27 (37.0%)	62 (40.3%)	22 (59.5%)	32 (50.8%)
Asian	1 (1.4%)	3 (1.9%)	0	1 (1.6%)
Cancer type				
HEC				
Breast	40 (54.8%)	—	20 (54.1%)	—
NSCLC	33 (45.2%)	—	17 (45.9%)	—
MEC				
Colorectal	—	93 (60.4%)	—	32 (50.8%)
Pancreatic	—	23 (14.9%)	—	12 (19.0%)
Gastric	—	13 (8.4%)	—	9 (14.3%)
Bladder	—	9 (5.8%)	—	5 (7.9%)
Appendix	—	9 (5.8%)	—	3 (4.8%)
Rectal	—	7 (4.5%)	—	2 (3.2%)
Chemotherapy				
HEC				
Doxorubicin/	40 (54.8%)	—	20 (54.1%)	—
Cyclophosphamide				
Cisplatin/pemetrexed	25 (34.2%)	—	9 (24.3%)	—
Cisplatin/paclitaxel	8 (11.0%)	—	8 (21.6%)	—
MEC				
FOLFOX	—	145 (94.1%)	—	58 (92.1%)
Gemcitabine/paclitaxel	—	9 (5.8%)	—	5 (7.9%)

Abbreviations: HEC, highly emetogenic chemotherapy; MEC, moderately emetogenic chemotherapy; NSCLC, non‐small cell lung cancer.

One hundred patients (*n* = 37 HEC and 63 MEC) experienced breakthrough CINV in Cycle 1 and were stratified to the NEPA‐based study treatments for Cycle 2 based on the chemotherapy emetogenicity (HEC and MEC).

Consistent with Cycle 1, the HEC group in Cycle 2 was comprised of predominantly females with breast cancer receiving AC chemotherapy (Table 1). The remainder of this group were patients with NSCLC receiving cisplatin‐based chemotherapy. The majority of the MEC group were male; Although there was a diverse group of tumor types, most patients had colorectal cancer and received FOLFOX chemotherapy.

### 3.2 Efficacy

#### 3.2.1 Cycle 1

**FIGURE 2:**
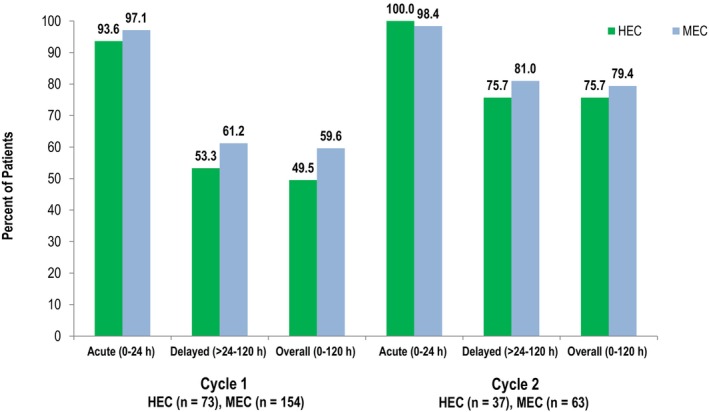
Complete response (no emesis/no rescue use) rates in Cycles 1 and 2.

Despite administration of guideline‐recommended prophylaxis prior to chemotherapy, overall complete response rates in Cycle 1 were quite low; 50% in the HEC group and 60% in the MEC group (Figure 2). The no nausea rates were similarly low; during the overall phase no nausea rates were 43% in the HEC group and 30% in the MEC group (Table 2).

**TABLE 2:** No nausea rates in Cycles 1 and 2.

Time interval	Cycle 1		Cycle 2	
	HEC (*n* = 73)	MEC (*n* = 154)	HEC (*n* = 37)	MEC (*n* = 63)
Acute (0–24 h)	90.4%	90.3%	100%	95.2%
Delayed (> 24–120 h)	45.2%	31.2%	70.3%	41.3%
	42.5%	29.9%	70.3%	36.5%
Overall (0–120 h)				

#### 3.2.2 Cycle 2

Despite the fact that all patients entering Cycle 2 had experienced breakthrough CINV in Cycle 1 and would therefore be at increased risk for CINV, the complete response rates were higher in Cycle 2 following the change to antiemetic prophylaxis (Figure 2). The complete response rates during the overall phase were 76% and 79% in the HEC and MEC groups, respectively (Figure 2, Table 3). There was essentially almost complete control of CINV during the acute phase in both groups, with any breakthrough CINV occurring during the delayed phase.

**TABLE 3:** Cycle 2 complete response rates with two‐sided 95% confidence intervals.

Phase	HEC (*N* = 37)	MEC (*N* = 63)
Acute (0–24 h)	100.0% (90.5, 100)	98.4% (91.5, 100)
Delayed (> 24–120 h)	75.7% (58.8, 88.2)	81.0 (69.1, 89.8)
Overall (0–120 h)	75.7% (58.8, 88.2)	79.4 (67.3, 88.5)

Although no nausea rates were also improved in Cycle 2 relative to Cycle 1, control of nausea remained challenging, particularly in the MEC group. During Cycle 2, no nausea rates during the overall phase were 70% in the HEC group and just 37% in the MEC group (Table 2). The nausea occurred predominantly during the delayed phase; the percentages of patients with no nausea in the acute phase were 100% and 95% in the HEC and MEC groups, respectively, whereas 70% and 41% of patients in the HEC and MEC groups had no nausea during the delayed phase. The higher no nausea rates in the HEC group are most likely due to the concomitant use of olanzapine. In the subset of patients who experienced nausea, the median nausea scores (on the scale from 0 to 10) were five in the HEC group and seven in the MEC group.

#### 3.2.3 Complete Response for Subgroups of Males and Females

Complete response rates were similar for males and females in the HEC and MEC groups during both Cycles 1 and 2 (Table 4).

**TABLE 4:** Complete response rates for males and females in Cycles 1 and 2.

Cycle/Phase	HEC		MEC	
Cycle 1	Males (*N* = 30)	Females (*N* = 43)	Males (*N* = 75)	Females (*N* = 79)
Acute (0–24 h)	94.5%	93.1%	97.1%	96.5%
Delayed (> 24–120 h)	52.3%	54.5%	62.3%	60.8%
Overall (0–120 h)	50.6%	48.7%	60.6%	58.9%
Cycle 2	Males (*N* = 12)	Females (*N* = 25)	Males (*N* = 37)	Females (*N* = 26)
Acute (0–24 h)	100%	99.1%	98.1%	99.1%
Delayed (> 24–120 h)	75.1%	77.2%	82.5%	79.3%
Overall (0–120 h)	74.3%	76.3%	80.3%	78.1%

### 3.3 Safety

There were no unexpected adverse events in patients receiving either NEPA plus olanzapine and dexamethasone or NEPA plus dexamethasone. There were also no serious adverse events and no adverse events leading to discontinuation of any patient.

## 4 Discussion

The ultimate goal of antiemetic guidelines is to provide direction to optimize prevention of CINV as, once established, it is very difficult to control. Although the antiemetic guidelines offer recommendations for managing breakthrough CINV [14, 17, 18], the recommendations are not very specific and include language such as “consider changing”, “consider adding”, “possibly switch,” and “possibly adjust” [18]. Additionally, minimal clinical evidence has been generated thus far to support a recommendation on how best to prevent CINV in the subsequent cycles after breakthrough CINV occurs.

In clinical practice patients experiencing CINV in the previous cycle are very often given the same antiemetic prophylaxis in the following cycles, exposing them to a high risk of repeatedly experiencing CINV [3]. Previous work has shown that patients who experience CINV in Cycle 1 are at increased risk for CINV in subsequent cycles [3, 35, 36]. In a post hoc analysis by Navari et al. [35], among the 99 patients treated with an NK1 RA regimen who experienced breakthrough CINV in Cycle 1, 55% experienced breakthrough CINV again in Cycle 2, 89% in Cycle 3, and 74% in Cycle 4. Similarly, in the trial exploring switching to granisetron extended‐release subcutaneous injection in Cycle 2 in the subset of patients who failed to achieve complete response in Cycle 1, a small group of patients (*n* = 30) continued to receive the same 5‐HT3 RA as in Cycle 1, despite experiencing breakthrough CINV; only 16% of these patients achieved a complete response in Cycle 2 [23].

Prior studies have looked at evaluating agents as rescue therapy to treat existing breakthrough CINV [11, 19, 20, 21]. However, to date, few studies have evaluated adding or changing agents in the subsequent cycle as suggested by the guidelines. Additionally, although multiple cycle antiemetic trials are relatively common, particularly when assessing the safety of new antiemetic agents, they consistently only report the population‐wide CINV rates across each successive cycle of chemotherapy without examination of the efficacy of antiemetics in the subset of patients with initial and potentially repeated breakthrough CINV. The current study was designed to ensure that patients in Cycle 1 were receiving guideline‐directed antiemetic prophylaxis based on the emetogenicity of the chemotherapy. This allowed for a within patient direct comparison when adding new agents in Cycle 2 after breakthrough CINV occurred in Cycle 1. Complete response, the standard clinical endpoint in antiemetic trials, was selected as the primary endpoint in this study.

As the newest NK1 RA in the class and as a unique NK1 RA/5‐KT3 RA fixed combination, IV NEPA was selected for use in this study. In vitro studies have shown that the combination of netupitant and palonosetron exhibits a synergistic effect in preventing the NK1 receptor response against its endogenous agonist, substance P [37]. The extended elimination half‐lives of netupitant (80 h with oral NEPA and 144 h with intravenous (IV) NEPA after conversion from fosnetupitant) and palonosetron (50 h) are believed to contribute to long‐lasting CINV protection [37, 38]. Registration studies showed oral NEPA combined with dexamethasone is more effective than palonosetron combined with dexamethasone in both the HEC setting and in patients receiving anthracycline/cyclophosphamide (AC) chemotherapy [26, 27, 28, 29]. The approval of IV NEPA was based on demonstrating the pharmacokinetic equivalence of IV fosnetupitant to oral netupitant [38]. In a Phase 3 study in patients receiving cisplatin‐based HEC, IV NEPA displayed similar safety to oral NEPA [39]. A follow‐up Phase 3b study in patients receiving AC‐based chemotherapy showed comparable efficacy for IV and oral NEPA [40]. Unlike some other IV NK1 RAs, IV NEPA did not cause any treatment‐related injection‐site AEs and there were no reports of hypersensitivity or anaphylaxis for IV NEPA [39, 40].

In this study, patients receiving HEC who developed breakthrough CINV after their first course of chemotherapy (and antiemetic prophylaxis of fosaprepitant/palonosetron/dexamethasone) had significant improvement in CINV control after the second course of chemotherapy when given NEPA plus olanzapine, and dexamethasone; impressively, 76% of all HEC patients with breakthrough CINV in Cycle 1 experienced complete response in Cycle 2 (in other words only 24% experienced breakthrough CINV). It is noteworthy that the overall complete response rate in Cycle 1 for patients receiving fosaprepitant/palonosetron/dexamethasone was 50%. Similarly, patients receiving MEC who received palonosetron plus dexamethasone in Cycle 1 had significant improvement in CINV control after the second course of chemotherapy when given NEPA and dexamethasone as antiemetic prophylaxis; 79% of all MEC patients with breakthrough CINV in Cycle 1 experienced complete response in Cycle 2 (in other words 21% experienced breakthough CINV). The overall complete response rate for the MEC group in Cycle 1 was 60%. The high response rates in Cycle 2 after changing/adding new NEPA‐inclusive antiemetics to the regimen given in Cycle 1 suggest that this was a population at high emetic risk, who may have benefitted from these regimens upfront in Cycle 1.

It is well‐established that nausea is harder to control than emesis [18] and though there is some evidence that NEPA may offer benefits over a 5‐HT3 RA plus dexamethasone in controlling nausea that are not consistently seen with other NK1 RAs [41], preventing nausea remains the greatest unmet need [42]. The NEPA/olanzapine regimen was clinically effective for the HEC patients for the secondary endpoint of “no nausea” for the 5 days post chemotherapy; however, there was a much lower rate of no nausea for MEC patients. Given that olanzapine has been shown to be particularly beneficial in providing nausea control [10, 11], this was likely a contributing factor for better nausea control in the HEC group, where olanzapine with administered with NEPA. As the complete response rate was 79% in the MEC group (which is the composite of no emesis and no use of rescue medication), a number of patients experiencing some level of nausea did not seek rescue treatment, suggesting that perhaps the nausea may not have been overly bothersome.

Limitations of this study include the fact that this study does not answer the question as to whether or how much changing the NK1 RA alone in Cycle 2 or adding olanzapine to the existing NK1 RA‐based regimen would have improved CINV control in the subsequent cycle because both modifications were made to the regimen in Cycle 2. A separate study or studies would need to be performed to evaluate the individual benefits of changing from a different NK1 RA to NEPA or adding olanzapine to the existing NK1 RA regimen; our study is only able to conclude the benefits of the change to the NEPA/olanzapine combination. As there is evidence suggesting that NEPA may provide better CINV control than an aprepitant‐based regimen during the latter part of the delayed phase (Days 3–5) [30, 31] and during the extended overall (0–168 h) phases in the HEC setting [32, 43], and as olanzapine has shown the greatest benefit in improving nausea control, switching from fosaprepitant to NEPA and adding olanzapine in the HEC setting following breakthrough CINV was thought to have the best chance of optimizing CINV control. Another limitation of this study was the absence of a blinded control group retaining the original treatment that patients received in Cycle 1. The inclusion of this control group would have allowed a direct comparison of CINV control to assess the benefits of the NEPA/olanzapine regimen. However, the response rates during Cycle 1 offer a reasonable control. When comparing the results between Cycles 1 and 2, it is important to keep in mind that patients entering Cycle 2 were at increased risk of CINV having experienced breakthrough CINV in Cycle 1. Another limitation of this analysis was the smaller (*n* = 37) than targeted sample size (*n* = 50) in the HEC group.

## 5 Conclusion

In conclusion, the results of this study evaluating changing the antiemetic regimen following breakthrough CINV demonstrate that the use of NEPA with or without olanzapine is a highly effective prophylactic approach for patients receiving HEC or MEC who develop breakthrough CINV after their first course of chemotherapy. The results support the current recommendations of the international antiemetic guidelines and offer specific evidence for adding new agents to the subsequent cycle of chemotherapy following breakthrough CINV.

## Author Contributions

**Rudolph M. Navari:** conceptualization (lead), data curation (lead), formal analysis (supporting), funding acquisition (lead), investigation (lead), methodology (equal), project administration (lead), resources (lead), software (supporting), supervision (lead), validation (equal), visualization (equal), writing – original draft (lead), writing – review and editing (equal). **Erminio Bonizzoni:** data curation (supporting), formal analysis (lead), investigation (supporting), methodology (equal), project administration (supporting), resources (supporting), software (lead), supervision (supporting), validation (equal), visualization (equal), writing – original draft (supporting), writing – review and editing (equal).

## Ethics Statement

The trial protocol was approved by the Simon‐Williamson Clinic Compliance/Institutional Review Board. All patients provided written informed consent prior to initiation of any study treatment, and the study was conducted in accordance with recognized international scientific and ethical standards, including but not limited to the International Conference on Harmonization guideline for Good Clinical Practice (ICH GCP) and the Declaration of Helsinki.

## Conflicts of Interest

Rudolph M. Navari declares no conflicts of interest. Erminio Bonizzoni has received consulting fees from Helsinn Healthcare and Zambon Biotech.

## Data Availability

The data presented in this study are available on request from the corresponding author. The data are not publicly available due to ethical regulation.
